# COMPARISON BETWEEN INCIDENCE OF INCISIONAL HERNIA IN LAPAROSCOPIC CHOLECYSTECTOMY AND BY SINGLE PORT

**DOI:** 10.1590/0102-672020180001e1354

**Published:** 2018-06-21

**Authors:** Fernando Athayde MADUREIRA, Cristiane Luzia Teixeira GOMEZ, Eduardo Monteiro ALMEIDA

**Affiliations:** 1 Postgraduate Program in General Surgery of the Federal University of Rio de Janeiro State; 2 Postgraduate Program in General Surgery of the Pontifical Catholic University), Rio de Janeiro, Brazil

**Keywords:** Incisional hernia, Cholecystectomy, laparoscopic, Minimally invasive surgical procedures., Hérnia incisional, Colecistectomia laparoscópica, Procedimentos cirúrgicos minimamente invasivos

## Abstract

**Background:**

Surgeries with single port access have been gaining ground among surgeons who seek minimally invasive procedures. Although this technique uses only one access, the incision is larger when compared to laparoscopic cholecystectomy and this fact can lead to a higher incidence of incisional hernias.

**Aim:**

To compare the incidence of incisional hernia after laparoscopic cholecystectomy and by single port.

**Methods:**

A total of 57 patients were randomly divided into two groups and submitted to conventional laparoscopic cholecystectomy (n=29) and laparoscopic cholecystectomy by single access (n=28). The patients were followed up and reviewed in a 40.4 month follow-up for identification of incisional hernias.

**Results:**

Follow-up showed 21,4% of incisional hernia in single port group and 3.57% in conventional technique.

**Conclusions:**

There was a higher incidence of late incisional hernia in patients submitted to single port access cholecystectomy compared to conventional laparoscopic cholecystectomy.

## **RESUMO**

**Racional:**

Operações com acesso por portal único vêm ganhando espaço entre cirurgiões que almejam procedimentos minimamente invasivos. Embora esta técnica utilize apenas um acesso, a incisão é maior quando comparada à colecistectomia videolaparoscópica e esse fato pode levar à maior incidência de hérnias incisionais.

**Objetivo:**

Comparar a incidência de hérnia incisional pós-colecistectomia videolaparoscópica e por portal único.

**Métodos:**

Cinquenta e sete pacientes foram divididos aleatoriamente em dois grupos e submetidos à colecistectomia videolaparoscópica convencional (n=29) e colecistectomia videolaparoscópica por acesso único (n=28). Os pacientes foram acompanhados e revisados por 40,4 meses para identificação de hérnias incisionais.

**Resultados:**

No seguimento a taxa de hérnia incisional encontrada no grupo de acesso único foi de 21,4% e de 3,57% no submetido à técnica convencional.

**Conclusões:**

Verificou-se maior incidência de hérnia incisional tardia nos pacientes submetidos à colecistectomia por portal único em relação à colecistectomia vídeolaparoscópica convencional.

## INTRODUCTION

The first open cholecystectomy was performed in 1882 by Carl Langenbuch in a 43-year-old male patient with symptomatic cholelithiasis^17^. More than 100 years later, in Germany, the first laparoscopic cholecystectomy was performed by Erich Mühe de Böblingen in 1985^24^, being modified by the French physician Phillipe Mouret in 1987, by the addition of support by micro camera, becoming the first video-laparoscopic cholecystectomy (CVL)^13^. Currently it is the gold standard for the treatment of cholecystolithiasis and its variations, being the most common indication for elective surgery^21^ ^,^ ^25^. The tendency is to make comparable to the gold standard the different types of surgical approaches that have one or no cutaneous incision. In this sense, the search for reducing the surgical impact, morbidities and complications justify the development of minimally invasive procedures^16^. Suggesting a lower inflammatory response, less pain in the postoperative period and better aesthetic result ^6^ ^,^ ^27^ ^,^ ^30^.

In single-portal or single-port (SP) operation, although it is a minimally invasive technique, a single surgical approach is used, with a larger aponeurotic opening than CVL, which may or may not increase the risk of incisional hernia lesions at this site^12^ ^,^ ^5^.

The objective of this study was to compare the incidence of incisional hernias in patients submitted to CVL and cholecystectomy by SP.

## METHOD

In a prospective randomized controlled study, 57 patients undergoing cholecystectomy were operated on in the 6^th^ ward of Clínica Cirúrgica A between August 2010 and July 2011 at the Gaffrée and Guinle University Hospital of the Federal University of Rio de Janeiro State, Brazil. Fifty-four women and three men, all randomly divided into two groups to undergo elective cholecystectomy. Among these, 29 performed conventional multi-portal CVL and 28 by SP. Only 56 patients were included in the study due to a death in the CVL group, due to an event not related to the study. All were followed at the outpatient clinic of the hospital about 40.4 months after the surgical procedure or contacted through active search and reassessed in the outpatient clinic.

Those older than 18 years before surgery, who had symptomatic or asymptomatic cholecystolithiasis, and gallbladder polyps larger than 1 cm were included. Those who presented acute cholecystitis, jaundice and pancreatitis at the preoperative evaluation and who had a scleroatrophic gallblader denoted by ultrasonography, were excluded.

Wound healing, stitch removal and complications such as infections, seromas, and hernias were evaluated at outpatient follow-up. All variables were recorded through their own data collection form. In order to analyze the similarity between the groups, the body mass index, age, gender, comorbidities and previous abdominal operation were considered^20^.

The type and size of the umbilical incision in the skin, the internal diameter of the aponeurosis, the total duration of the operation in minutes and in each step were recorded (trocar placement time, bed vesicle detachment, synthesis and effective operation time). The size of the umbilical incision and internal diameter of the aponeurosis were evaluated at the end of the procedure using a sterile compass to measure the distance (vertical) between the two opposite sides of the incision. The synthesis of aponeurosis in both groups was performed with continuous suture using Vicryl® number 0

### Statistical analysis

The data collected from the two groups were compared using the mean or the median as a measure of central tendency. From the quantitative variables, the Student t test was used to compare means, and the Wilcoxon signed rank test to the medians. Chi-square, or Fisher’s exact test, was used to analyze qualitative variables. The p<0.05 was considered statistically significant. The sample calculation was done for the inflammation markers dosed at the time, considering a difference of up to 35% as expected between the two groups. The margin of error was 5% (p=0.05), and the study’s “Power” was 80%. The sample calculation provides a sample of 19 patients on each side. At the time of the study design, there was no similar work published and the theoretical value of 35% was adopted from similar studies. For data computation, the Excel^®)^ and Word^®)^ programs of the Microsoft Office 2010^®)^ package were used, as well as the IBM SPSS^®)^ version 22.

## RESULTS

Fifty-six patients with a mean follow-up of 40.4 months were followed. The characteristics of the study population as age, body mass index, comorbidities and previous abdominal surgery are shown in Table 1.

**TABELA 1:** Characteristics of patients

	CAP	Comorbidities	Age	BMI
SP	8	8	48.64 (27 - 67)	28.15 (24 - 41.9)
CVL	17	18	64.67 (33 - 90)	24.86 (16 - 34.52)
p	.064	.008	>.001	.138

CAP=previous abdominal surgery; BMI=body mass index

**FIGURE 1:**
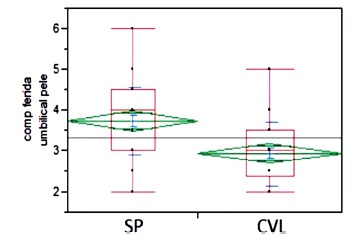
Umbilical incision of the skin

**FIGURE 2:**
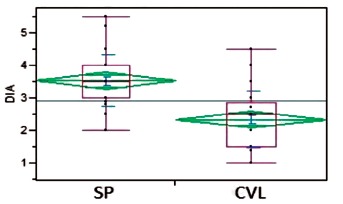
Distribution of abdominal diameter

The mean internal diameter of the aponeurosis was in the SP group of 3.6 cm and in the CVL 2.3 cm (p<.0001). The umbilical incision of the skin in the SP group was on average 3.7 cm and 2.9 cm in the CVL group (p<.0001). (Figures 1 and 2)

SP=single port; CVL=videolaparoscopic cholecystectomy

SP=single port; CVL=videolaparoscopic cholecystectomy

There was no statistical difference for the average duration of operations or stages of operation between the two groups (Table 2). Seven patients progressed with incisional hernia, six of which belonged to the SP group (21.4%) and one from the CVL (3.6%, p=0.012, Table 2).

**TABLE 2:** Surgical results

	SP (n=28)	CVL (n=28)	p
Umbilical incision ^a^	3,7 (2,0-6,0)	2,9 (2,0-5,0)	<,0001
Abdominal diameter ^a^	3,6 (2,0-5,5)	2,3 (1,0-4,5)	<,0001
Surgical time ^b^	60,3 (32-128)	51,3 (25-120)	0,11
Effective surgical time ^b^	34,9 (10-61)	29,08 (7,0-65)	0,19
Removal time ^b^	8,3 (2,0-20)	7,23 (2,0-30)	0,85
Closing time ^b^	8,21 (2,0-9,0)	9,14 (3,0-15)	0,08
Seroma	8%	12%	0,32
Wound infection	2,8%	2,9%	0,9
Gallbladder perforation	15.69%	5,88%	0,028
Late incisional hernia	6	1	0,012

a Incision and diameter (cm); ^b^ Time (min)

## DISCUSSION

It was observed that the incidence of incisional hernia in the two groups presented statistical difference (p=0.012). In the CVL group there was only one case of late incisional hernia (3.57%), and in SP, six cases (21.4%). In the CVL group, the only case of hernia is an 85-year-old woman with a body mass index of 21.48 kg/m^2^, diabetic, hypertensive and not submitted to previous abdominal operation. From the SP group, five patients with hernia had no comorbidities and were not submitted to previous abdominal operation. Only one patient in the SP group with a hernia was hypertensive and submitted to tubal ligation 30 years ago. These patients have ages between 28-67 years, body mass index between 25-41.92 kg/m^2^.

Analyzing risk factors such as infection, wound complication and body mass index, there was no statistical difference. The wound infection rate was 2.8% on average in the SP group and 2.9% on average in the CVL group (p=0.9).

The mean body mass index was 24.86 kg/m^2^ in the CVL group, and in the SP it was 28.15 kg/m^2 )^(p=0.138), which in theory could influence the final result of the hernia occurrence.

The age variable did not influence the onset of hernias. The average of SP group was 48.64 years and in the CVL 64.67 years (p>0.001).

The single umbilical access for cholecystectomies intend to have an excellent esthetic effect, with only one site of pain, low potential of infection and low morbidity^20^ ^,^ ^29^. The late finding of incisional hernias, however, questions these benefits and questions the validity of this technique^20^.

Husnu A, et al. followed, a group of 163 patients; 111 were submitted to CVL (13-21 months) and 52 did videolaparoscopic cholecystectomy with a single portal (10-20 months). Two (1.8%) had trocar site hernia in the CVL group and three (5.8%) in the CVL group with a single portal. Statistically according to the study, incisional hernia after CVL with a single portal is more frequent than reported in the literature^1^.

Gangl et al. in a retrospective study compared the incidence of hernias in 67 patients submitted to CVL and 67 by SP in the same period, in a follow-up of 17-26 months. The incidence of late incisional hernia was 1.9% (n=1 from 53 patients) in the group undergoing SP and 2.1% (n=1 from 48 patients) in the CVL group, indicating that there was no significant difference in the used technique^8^.

Antoniou did in 2011 a systematic review of the specific literature covering a total of 1156 patients undergoing cholecystectomy by SP, accounting for 3.6% (n=40) of them with incisional hernia. This article suggests caution in patients with advanced age but demonstrated clinically satisfactory results^3^.

In a prospective and multicenter study published in the Journal of the American College of Surgeons, Jefrey Marks found a higher incidence of hernias in the SP (p=0.03). This study concludes that the method is safe, but that the aesthetic effect still overlaps the hernia rate^22^. Another study corroborates the finding of a higher incidence of incisional hernia in SP surgeries^1^.

Antoniou SA et al. in 2015, analyzed 19 randomized trials covering 1705 patients submitted to conventional videolaparoscopy and SP; 0.7% and 2.2% of the patients presented hernia at the trocar site respectively (p=0.05)^2^.

Christoffersen MW, et al., in a cohort study based on prospective data between 2009 and 2011, 552 patients (SP, n=185 and CVL, n=367) were evaluated with mean observation time of 48 months. The hernia rate at the trocar site was 4% in SP and 6% in CVL (p=0.560). The result did not indicate a significant difference in the incidence of incisional hernia between groups^7^.

Sulu B, et al, selected 60 patients undergoing elective cholecystectomy by CVL and SP with 30 patients in each group. Two in the SP group had hernia at the trocar site in a follow-up of 12-20 months, concluding that these patients need a longer follow-up^28^.

The incidence of incisional hernia in this study was higher than expected, it may be questioned that the transumbilical localization of SP, which causes an incision of the aponeurosis larger than in the CVL and located in the alba line, often in patients with diastasis. It is also worth investigating whether transverse incisions and in another topography would also lead to this result.

Another point to be analyzed is whether the synthesis of aponeurosis that was performed in both groups with continuous suture could have influenced these results. Perhaps separated stitches or non-absorbable threads can be studied in future.

## CONCLUSION

The incidence of incisional hernia was higher in patients operated on for cholecystectomy by single port than in patients operated by traditional laparoscopic cholecystectomy with multiple portals.
